# Astroglial Isopotentiality and Calcium-Associated Biomagnetic Field Effects on Cortical Neuronal Coupling

**DOI:** 10.3390/cells9020439

**Published:** 2020-02-13

**Authors:** Marcos Martinez-Banaclocha

**Affiliations:** Department of Pathology, Lluis Alcanyis Hospital, Xátiva, 48006 Valencia, Spain; martinez_marben@gva.es

**Keywords:** astrocyte, magnetic field, electric field, ephaptic, coupling, calcium, connexin, isopotentiality

## Abstract

Synaptic neurotransmission is necessary but does not sufficiently explain superior cognitive faculties. Growing evidence has shown that neuron–astroglial chemical crosstalk plays a critical role in the processing of information, computation, and memory. In addition to chemical and electrical communication among neurons and between neurons and astrocytes, other nonsynaptic mechanisms called ephaptic interactions can contribute to the neuronal synchronization from different brain regions involved in the processing of information. New research on brain astrocytes has clearly shown that the membrane potential of these cells remains very stable among neighboring and distant astrocytes due to the marked bioelectric coupling between them through gap junctions. This finding raises the possibility that the neocortical astroglial network exerts a guiding template modulating the excitability and synchronization of trillions of neurons by astroglial Ca^2+^-associated bioelectromagnetic interactions. We propose that bioelectric and biomagnetic fields of the astroglial network equalize extracellular local field potentials (LFPs) and associated local magnetic field potentials (LMFPs) in the cortical layers of the brain areas involved in the processing of information, contributing to the adequate and coherent integration of external and internal signals. This article reviews the current knowledge of ephaptic interactions in the cerebral cortex and proposes that the isopotentiality of cortical astrocytes is a prerequisite for the maintenance of the bioelectromagnetic crosstalk between neurons and astrocytes in the neocortex.

## 1. Introduction

In addition to chemical and electrical neurotransmission, nonsynaptic mechanisms known as ephaptic interactions [1,2] are considered critical for the synchronization of neurons into the neocortex in physiological and pathological conditions [3,4,5,6], playing a central role in cognitive faculties [7,8]. The integration of synaptic currents, action potential currents, and astroglial ionic currents contribute to the ephaptic effects in the neocortex through the modulation of the extracellular local field potential (LFP) [9,10,11]. The effects of the action potentials and astroglial ionic currents have been considered negligible in comparison to the synaptic currents [12,13]. Therefore, the role of astroglial bioelectric fields on the LFPs has been scarcely studied. However, there is increasing evidence that bioelectromagnetic fields generated by the astroglial syncytium around neurons may contribute to the modulation and synchronization of neuronal behavior in the neocortex. These effects may help to explain, at least partially, the therapeutic actions of transcranial electric and magnetic field stimulation [14].

## 2. The Complexity of the Neocortex

**Figure 1:**
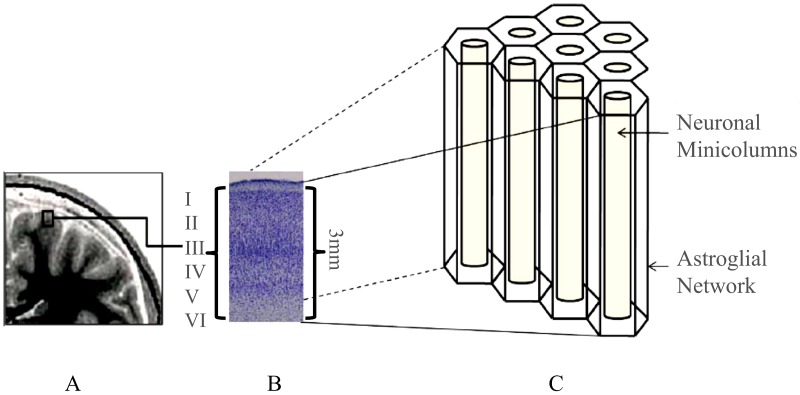
Three-dimensional (3D) organization of the human neocortex. Scanning imaging of the human brain (**A**). Histological section of the human cerebral cortex organized in layers (**B**). Astroglial matrix (hexagonal network) surrounding neuronal minicolumns and layers (**C**).

Knowledge of the complexity of the functional architecture and connectivity in the mammalian cerebral cortex began with Cajal’s studies [15], and have not finished yet [16]. The neocortex is composed of neurons and glial cells organized in two closely related 3D structures (Figure 1). The neocortex is organized in neuronal layers and columns [15,16,17,18,19,20,21], whereas the gliocortex is composed of highly organized astrocytes forming a 3D complex syncytium [22], which complement neocortex working as a functional matrix [23,24].

**Figure 2:**
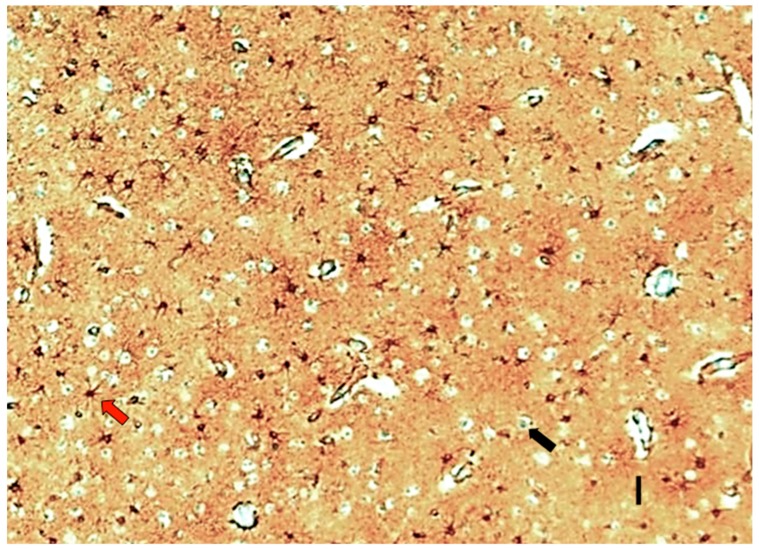
Histological section of the human neocortex (layers 2–3) parallel to the pial surface (scale bar, 50 μm). Astrocytes (red arrow) were immunolabeled with GFAP (glial fibrillary acidic protein). Body neurons (black arrow) were not labeled. Astroglial matrix is a sponge-like 3D-structure where neurons of the cortex are imbibed.

The complexity of astrocytes in the neocortex has increased across evolution, likely supporting the organization of neuronal circuitries and the development of superior cognitive faculties [25]. Astrocytes occupy a large volume in the neocortex, and their branches concentrate around neuronal synapses in a specific manner, constituting around 20% of the total cell number in most adult brain regions [25]. Each astrocytic domain can integrate and modulate the information processing of thousands of synapses under discrete and specific neocortical regions [26] (Figure 2). A single astrocyte in the human cerebral cortex may cover between 2.7 × 10^5^ to 2 × 10^6^ synapses within a single domain [27,28,29,30]. Interestingly, astrocytes from layers 2 and 3 of the neocortex are greater and occupy a large volume than astrocytes from layers 4–6 and layer 1, which result in more extensive coverage of synapses in layers 2 and 3 [31]. Moreover, in primates, intralaminar and interlaminar astrocytes extend vertical processes that traverse several cortical layers and run parallel to apical dendrites within the supragranular neocortex (above layer 4). This structure of the astroglial network provides an extra-organization of the supragranular neuropil, enclosing neuronal minicolumns [28,32].

Although only the peripheral astrocytic processes interconnect through gap junctions allowing the movement of ions among astrocytes forming a functional syncytium [22,33], these cells are highly organized, exhibiting a close functional dialogue with neurons [34] participating in the modulation of slow oscillations [35]. Astrocytes can change the membrane expression of ion channels and neurotransmitter receptors following sustained afferent inputs [36]. Furthermore, imaging studies showed that thin astrocytic processes change their shape at a time scale of a few minutes in response to long-term potentiation (LTP) protocols [36,37]. These structural changes are an expression of the high astroglial plasticity, which correlates to the synaptic changes, supporting the close molecular neuron–astroglial crosstalk. This continuous, bidirectional, and plastic neuron–astroglial dialogue likely contributes, in a more realistic way, to cognitive faculties.

### Structural–Functional Relationship in the Neocortex

Cognitive faculties rely on the cerebral cortex, a complex structural and functional region of the brain that contains the discrete elements that support information processing and computation. However, specific cognitive functions are associated with the coordinated activation of different brain regions [38]. Although we know the structural interconnectivity among the different brain regions, we do not understand the fundamental mechanisms that drive the coordinated functional interplay among specific brain regions and how this interplay results in the expression of different cognitive processes.

The use of tractography and constructed structural brain networks in healthy humans have shown a hidden distributed and overlapping control architecture that brings high efficiency in the transition among network states supporting cognitive functions as well as giving robustness against damage [38]. This hidden brain structure seems to be supported by a minimal subset of dominating nodes that control the remaining nodes with one-step direct interaction [38]. Moreover, a recent computational approach using magnetic resonance imaging (MRI) provided evidence that effective connectivity within the entire brain connectome during a specific language-processing task increases from childhood to adulthood as a consequence of white matter maturation [39]. These results suggest that the maturation of glial cells is at least as necessary as the establishment and consolidation of neuronal circuits and their corresponding synapses. Specifically, astrocytes of the cerebral cortex seem to play a critical role in the processing of information and can contribute, among other well-established mechanisms, to the synchronization of neuronal activities between different cortical areas. Accordingly, algebraic topology analysis of the direction of the synaptic connectivity in neocortical microcircuits has shown the emergence of groups of neurons that bound into cavities that seem to guide the emergence of neocortical activity [40]. This close relationship between the neocortical flow of information into correlated activity and its microcircuit architecture is consistent with the previous hypothesis about how information is processed and structured across the cerebral cortex [41].

Cognitive brain dynamics are associated with intricate activity patterns that seem spontaneously generated through a continuous and widespread change of activities among different brain regions [42]. Functional magnetic resonance imaging (fMRI) studies have shown different patterns of interaction in different conscious states. Indeed, in wakefulness, a global integration of brain activity predominates and simultaneously engages different cerebral areas [42]. Healthy conscious patients performing mental tasks showed complex and dynamic patterns of coordinated fMRI, whereas anesthesia increased the probability of less complicated functional patterns [42]. Surprisingly, patterns with high and low coordination and high modularity were associated with conscious cognition, which showed low similarity to anatomical connectivity. Contrarily, a pattern of low interregional dynamic coordination, low efficiency, and high similarity to anatomical connectivity was associated with reduced or even absent conscious cognition [42]. These results agree with previous models that suggest that alternating patterns of correlations between states of high and low connectivity are a fundamental property of information processing and cognitive function in the human brain [43]. Therefore, though there is a link between anatomical connectivity and diverse behavioral and cognitive tasks, recent fMRI studies show “spontaneous” changes reflecting an unknown coordination mechanism that underlines the functional brain dynamics [44].

Various models to explain synchronous communication between different cortical areas seem to overcome the mentioned structural and functional shortcomings. For example, a mechanism for the propagation of synchronous spiking activity within weakly coupled areas is the feeding forward networks (FFNs) based on the presence of resonance in excitatory/inhibitory networks into the neocortex [45]. This model is supported by a mechanism that emerges from the interaction between excitatory and inhibitory neurons in each FFN layer, which can be gradually amplified in every layer resulting in synchronic propagation among different cortical areas [45].

We propose an alternative explanation for the previous finding based on the conjecture that the hidden control of superior brain functions remains on a continuous and bidirectional bioelectromagnetic crosstalk between the astroglial matrix, and different but specific neuronal circuits from widespread cerebral regions [23,24,46,47,48].

## 3. Ionic Neuron–Astroglial Crosstalk in the Neocortex

The close relationship between neurons and astrocytes in the cerebral cortex work at several organization levels, supporting the notion that neuron–astroglial dialogue plays a central role in information processing and computation [28,33,49,50,51,52,53]. Indeed, disruption of the neuron–astroglial interplay has pathological consequences [54,55].

Neocortical activity results from the synchronization of thousands of individual neurons that work in sophisticated and very organized oscillations with specific frequency bands [56]. Experimental data and theoretical approaches support the idea that these oscillations are the result of many convergent molecular and electrophysiological mechanisms that critically merge from the neuron–astroglial close interplay [49,51,56]. All these mechanisms can be finally dependent on the high excitable properties of neurons and astrocytes into the neocortex that continuously receives and emits information. However, the excitability of cortical astrocytes and their crucial role in supporting neuronal activity and synchronization has been seriously considered in recent years. Therefore, it is essential to understand the excitable properties of neurons and astrocytes, which depends principally on ionic currents through membranes and the consequent modification of the corresponding LFPs [5,57].

### 3.1. Potassium

Extracellular K^+^ concentration (around 3 mM) is critical to maintaining the resting membrane potential on neurons and astrocytes [56] supporting, on the one hand, the neuronal excitability and activity and, on the other hand, the K^+^ clearance from the extracellular space by cortical astrocytes, playing a critical role in the modulation of neocortical oscillation at different frequencies [56]. Astrocytes take up the excess K^+^ in the extracellular space following a coordinated cycle associated with neuronal activity. Each astrocyte reuptakes K^+^ excess and immediately redistributes it through gap junctions to regions where intracellular K^+^ levels are low. This redistribution also results in astroglial ionic currents in those neighboring areas associated with activated neuronal circuits, showing three periods, the “baseline” period with extracellular K^+^ concentrations around 3 mM, the “high” K^+^ concentration period with increased levels of extracellular K^+^ as a consequence of neuronal spiking activity, and the “recovery” period when high extracellular K^+^ levels are washing into astrocytes [56]. Interestingly, the blockage of astroglial K^+^ uptake increased the resonance frequency range of neurons as well as the oscillation power at beta and gamma frequencies during “high” and “recovery” K^+^ periods [56]. Besides, the use of antagonists of Cx-43 increased the duration of the recovery period, boosting the maximal spiking frequency and enhancing the resonance frequency range resulting in increased power in the beta and gamma range during the “high” K^+^ period. Besides, there was an increase in the power of the majority of oscillation frequencies during the “recovery” period [56]. Although gap junction blocking can also interfere with Ca^2+^ waves through the astroglial matrix, these results show the importance of K^+^ homeostasis in the astrocytic modulation of cortical coupling at different frequencies.

### 3.2. Calcium

Considering astrocytes as modulators of the synaptic activity into the neocortex is central to understanding what function astroglial Ca^2+^ transients may have in the cortex. These Ca^2+^ signals can, therefore, be activated in response to neuronal circuit working [58] or spontaneously by intrinsic mechanisms [59], though there are controversial results probably due to differences in the experimental settings [60]. Moreover, Ca^2+^ spontaneous events in astrocytes from layer 1 are different from those of layers 2 and 3, showing that the frequency and amplitude of Ca^2+^ transients are different though the astrocytic membrane potential was similar for astrocytes in all layers [37].

In primates, the primary visual cortex (V1) is organized in vertical columns according to preferred orientation maps, and astrocytes show robust and highly tuned Ca^2+^ responses to visual stimuli [60]. Regarding astrocytic Ca^2+^ transients secondary to neuronal working, experimental data suggest that a minimum amount of neuronal synaptic activity must reach the threshold associated with astroglial Ca^2+^ responses in vivo, suggesting that astrocytes are not involved in the millisecond level of perception as neurons are [60]. However, during spontaneous brain activity associated with anesthesia, whisker stimulation in the barrel cortex of mice showed that Ca^2+^ elevation in the perisynaptic astrocytes that surround thalamocortical axons consistently precedes local presynaptic neuronal Ca^2+^ signals [61]. Although the mechanisms are not understood, it seems that local astroglial Ca^2+^ signals participate, at least partially, in the modulation of spontaneous Ca^2+^ influx in neuronal presynapses. In the ferret visual cortex, the astrocytic subdomains respond independently to visual stimulus orientations by increasing cytosolic Ca^2+^ concentration, which is likely associated with the integration of local neuronal activities from multiple synapses at a spatial scale of 5–10 mm [62]. These results are in agreement with the concept that Ca^2+^ signals in astrocytes can integrate, with high spatial sensitivity and specificity, the visual information that activates hundreds of local neurons with thousands of synapses.

Finally, the role of astrocytic Ca^2+^ effects in neurovascular interaction was demonstrated with functional magnetic resonance imaging (fMRI) studies [63]. Diverse evoked Ca^2+^ responses in astrocytes were coupled with positive blood-oxygen-level-dependent (BOLD) signals, while intrinsic astrocytic Ca^2+^ signals coupled with negative BOLD signals. Both evoked and intrinsic astrocytic Ca^2+^ waves could occur concurrently or respectively during stimulation, but the evoked astrocytic Ca^2+^ signal only was detected at the activated cortical region whereas intrinsic astroglial Ca^2+^ transients were detected globally in multiple cortical regions [63]. Unlike propagating Ca^2+^ waves in spreading depolarization/depression, the intrinsic Ca^2+^ responses coincided in both hemispheres and were initiated upon activation of the central thalamus and midbrain reticular formation. The occurrence of the intrinsic astrocytic Ca^2+^ signal was strongly coincident with an increased electroencephalography (EEG) power level of the brain resting-state fluctuation. These results demonstrate highly correlated astrocytic Ca^2+^ spikes with bidirectional fMRI signals based on the thalamic regulation of cortical states, depicting brain state dependency of both astrocytic Ca^2+^ transients and BOLD-fMRI signals [63].

### 3.3. LFPs in Neocortical Synchronization

Neocortical oscillations are the result of rhythmic fluctuations of synchronized neuronal potentials belonging to specific neuronal circuits. These oscillations have a wide range of frequencies that are associated with specific states of consciousness and cognitive faculties, experimentally recorded as LFPs [56]. Since the excitability of neurons depends on the conductance of ions through cytoplasmic membranes induced principally by postsynaptic potentials, the addition of ephaptic potentials associated with neighboring LFPs may contribute to the subthreshold membrane potentials modulating the oscillation coupling of neurons [64].

Therefore, neurons in the neocortex usually work synchronously, generating extracellular currents that modulate the excitability of neighboring cells by virtue of the potential difference between LFPs associated with endogenous electrical activities. Although these field effects are feeble, in vitro experiments showed that little external applied fields were able to change the timing of spike activity [65]. In addition, this effect seems higher in oriented cortical structures like neocortex and hippocampus, where pyramidal cells organize in minicolumns with well-developed layers [66,67,68]. These particular arrangements of neurons in the cerebral cortex and hippocampus allow a parallel and radial alignment (orthogonal) of the interstitial space that has relatively low impedance to the extracellular ionic currents. Another example supporting ephaptic interactions is the arrangement and distribution of neurons in the specialized structure formed by the densely packed axons of basket cells around the Purkinje initial axon segment, which shows an ephaptic inhibition at very weak fields [69]. Also, the activity of a single Purkinje cell can contribute to LFPs sufficient to open sodium channels in neighboring Purkinje neurons allowing its synchronization [68].

### 3.4. LFP and Ca^2+^ Transients in Astrocytes

In vitro experiments demonstrated that slow oscillations (<1 Hz) with speeds around 0.1 m/s [49,51] are propagating waves that do not need chemical or electrical synaptic participation in the hippocampus, suggesting that ephaptic mechanism participates in the propagation and synchronization of these slow oscillations in the neocortex [70]. Some authors suggested that these types of slow oscillations derive from a single oscillator outside the hippocampal network [71]. Realistic cell models studying astrocytes from the CA1 hippocampal area predict that K^+^ intake via Kir4.1 generates small membrane depolarization across the astrocyte, suggesting that transients of extracellular K+ levels induce small changes of intracellular K^+^ concentrations that dissipate quickly due to very efficient K^+^ efflux [72]. Moreover, small changes in Ca^2+^ buffering may influence the spread of regenerative glial Ca^2+^ waves, being consistent with the slow Ca^2+^ transients experimentally reported [72]. In agreement, some models of astrocyte behavior have shown that astroglial Ca^2+^ waves may induce neuronal synchronization and spatial clustering of synaptic plasticity, showing that the number of inhibitory terminals that one astrocyte ensheathes is critical for the spatial extent and temporal duration of astrocytic Ca^2+^ transients [73]. However, when the number of active synapses increases into an astrocytic domain, there were simultaneous Ca^2+^ elevations in multiple microdomains that together were able to trigger the Ca^2+^ elevations in the thicker astrocytic branches, which were spatially expanded and prolonged in time having a lower frequency [73].

Previous data show that spatiotemporal patterns of Ca^2+^ fluctuation into neocortical astrocytes are intricate [74], even more so when Ca^2+^ waves propagate through the astroglial syncytium in physiological conditions [74]. Ca^2+^ transients spread intercellularly through gap junctions organized in different spatiotemporal events that participate in the modulation of neuronal coupling, which is crucial for the processing of information, including neuronal plasticity, learning, and memory [37]. For example, norepinephrine (NE) can decouple synchronized neuron–astroglial activity, inducing an increase in Ca^2+^ signaling into astrocytes and a simultaneous reduction in the spontaneous activity of neurons [75]. This inhibition of synchrony in neuronal activity might be associated with the regulatory effect of NE on sleep–wake cycles since slow-wave oscillations associated with sleep are highly synchronized, and they are the result of neuron–glial communication initiated by astrocytes [75].

### 3.5. Neocortical Astroglial Isopotentiality

The astroglial syncytium of the CNS is a sponge-like 3D-structure where neuronal circuits of the neocortex are imbibed. Then, neuronal transmission in each circuit seems sufficiently isolated from the interference of neighboring neurons. In this regard, neuronal circuits are like 3D-impressed circuits composed by discrete cellular elements interconnected by synapsis but imbibed in a matrix of astrocytes joined by gap junctions.

Neocortical astrocytes may drive a spatial buffer current by the movement of K^+^ ions from active sites to distant regions through gap junctions [76]. With some peculiarities in the hippocampus [76], astrocyte K^+^ currents can travel efficiently through neighboring astrocytes allowing a robust bioelectrical coupling among astrocytes that form part of the neocortical syncytium [77]. Therefore, the ability to equalize K^+^ extracellular concentration under syncytial isopotentiality is performed with more efficiency than previously thought [77,78], maintaining a sustained driving force for the efficient clearance of local increases of K^+^ concentration produced as the result of local neuronal activity, which in turn maintain the isopotentiality. Since each astrocyte directly couples to 7–9 astrocytes [77,78] by gap junctions, there is an excellent equalization of the astroglial membrane potential among those coupled cells.

Due to a high-density expression of K^+^ conductance and minimal Na^+^ and Ca^2+^ conductance through astrocytic membranes [78], the transmembrane potential of individual astrocytes cannot deviate substantially from each other, maintaining a quasi-physiological level of around −70 mV [77,78]. Efficient astroglial isopotentiality may facilitate the transients of different types of Ca^2+^ waves that propagate through the gap junction reaching distant astrocytes [76], suggesting a new intercellular transmission of information into the neocortex [76,77,78] that may also participate in astroglial coupling in conjunction with K^+^ and glutamate homeostasis [78]. Moreover, astroglial Ca^2+^ waves can modulate the interstitial LFPs contributing to the synchronization of cortical neurons at specific frequencies [24].

### 3.6. Mechanisms of Synchronization in the Neocortex

Some mechanisms have been proposed to explain the coupling of local and remote oscillations, but the exact nature of these processes remains unknown [45]. For example, gamma range oscillations (30–70 Hz) seem to facilitate the propagation of synchronous spiking neurons in weakly connected neuronal networks, providing temporal windows of excitability among different brain areas even though they were weakly connected [45]. This type of wave propagation needs to be coherent to allow the synchronous activity generated both by local circuits as well as by spatially distant neuronal circuits, which have to oscillate with matched phase and frequency.

One of the proposed mechanisms of synchronization is the communication through resonance that suggests that oscillations in each neuronal layer are driven by the waves in previous layers being naturally coherent in-phase [45]. In this regard, a recent paper has proposed that some chimeric states in neuronal networks may explain synchronization between different areas by bioelectromagnetic field coupling [79]. In nonlinear dynamics, a chimera state is the spatial concurrence of coherent (synchronous) and incoherent (nonsynchronous) dynamical behavior that arises in a network of oscillators [79]. This recent approach suggests that magnetic flux across the membrane of neurons can effectively act as a supplementary mode of information exchange, supporting the idea that alternating chimera patterns can emerge in a network of neuronal systems coupled through electromagnetic fields [79]. However, this mechanism cannot fully explain how spatially distant oscillators—belonging to any external or internal stimuli and those oscillations generated spontaneously—can synchronize in time, giving entrainment of coherence between areas.

Chemical neurotransmission is slow (1–2 ms between presynaptic and postsynaptic action potential) but more stable than bioelectromagnetic communication, which may be formed by three subcomponents: a direct electrical transmission between neurons through gap junctions; an ephaptic effect as the consequence of extracellular electric fields generated by neuronal activity; and, an ephaptic biomagnetic induction effect associated with astroglial Ca^2+^ ionic currents [23,24,46,47,48]. The latter component may constitute the external oscillator that, adjacent to neuronal networks in the hippocampus and other cortical areas, may connect cortical and subcortical regions, supported by the isopotentiality of the astroglial matrix. This biomagnetic component of the ephaptic coupling of neocortical neurons can depend on the astroglial isopotentiality that allows the preferential synchronization of slow oscillations. This mechanism of slow synchronized transients in hippocampal and cortical neurons may be explained by the modulation of astroglial biomagnetic fields on the extracellular local magnetic field potentials (LMFPs) mediated by specific Ca^2+^ waves through gap junctions in the astroglial matrix [5,6,24,70]. Therefore, we propose that astroglial isopotentiality may provide a biologically natural scaffold that contributes to the synchronization of neuronal activities from different cortical regions through ephaptic bioelectromagnetic interactions between the astroglial matrix and widespread neuronal circuits [23,24].

## 4. Bioelectromagnetic Fields in the Neocortex

There is a high correlation between EEG and magnetoencephalography (MEG) [80], though MEG records coherent magnetic fields from cortical pyramidal cells [81] resulting principally from the coordinated activity of neurons located in layers III and IV of the neocortex [82,83]. The magnetic fields recorded above the surface of the scalp correspond to those generated by neurons oriented parallel to the scalp surface with amplitudes of 25 to 100 nT at 1 mm, which may have enough biological effects [84].

**Figure 3:**
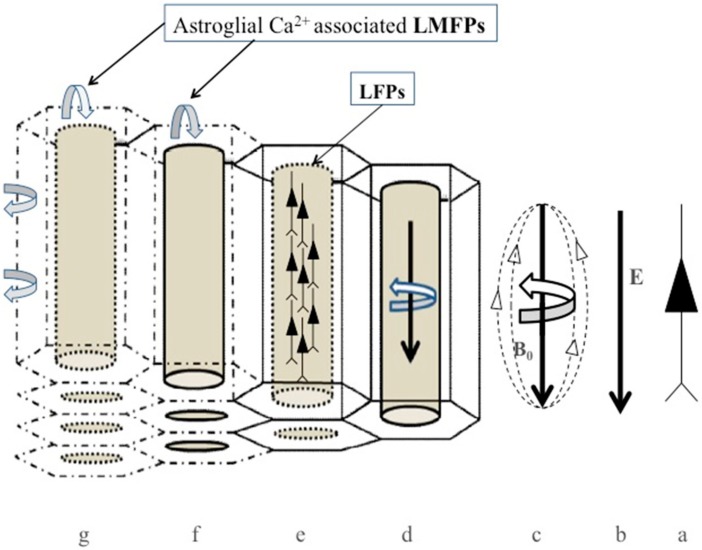
Schematic representation of the bioelectromagnetic field distribution in the neocortex and gliocortex. Schematic representation of a pyramidal neuron of the cerebral cortex (**a**). Bioelectric (E) and biomagnetic (B_0_) fields generated following the dendritic–axonal direction (**b** and **c**, respectively). Cylinders represent neuronal minicolumns, and hexagons represent the astroglial matrix (**d**–**g**). Bioelectromagnetic fields associated with neuronal activities in minicolumns (**d**), which modulate local field potentials (LFPs) (**e**). Ca^2+^-associated bioelectromagnetic fields can modulate LMFPs (**f**). Neuron–astroglial biomagnetic crosstalk (**g**).

The bioelectromagnetic fields in tissues dependent on the movements of ions across cells and cellular membranes [85], but their complexity increases because of the intricate structural organization of the neocortex. Therefore, the orientation of neurons and their anatomical relationship with astrocytes in the human neocortex is a critical factor determining the effects of endogenous bioelectric and biomagnetic field interactions (Figure 3). Since very narrow interstitial spaces separate neurons and astrocytes, the bioelectric and biomagnetic fields produced by neuronal currents can potentially modulate inactive adjacent cells (neurons and astrocytes) by ephaptic interaction in the adequate intracortical topology. The interstitial space into the neocortex is a very narrow and tortuous space among the cytoplasmic membranes of neurons and astrocytes that ranges between 20 to 64 nm [21,86,87]. It occupies approximately 15% of the total brain volume [88], suffering significant changes under physiological and pathological conditions [89,90], being electrically highly inhomogeneous [91].

Recent theoretical approaches have shown that neuronal electrical activities generated by the movement of ions through membranes modulate not only the synaptic communications but also result in time-varying electric fields and associated magnetic fields in virtue of Maxwell’s laws (Figure 3). These magnetic fields around neurons may be the ground of complex information processing in the cortex, and they may transfer and synchronize neuronal information [92]. Interestingly, spontaneous electromagnetic induction may have effects on the evolution of the self-organization of neuronal networks [93]. The magnetic coupling between neurons can contribute to the signal exchange of information among thousands of cells to promote the widespread synchronization of neuronal activities within a plastic structure as the neocortex [93]. Moreover, magnetic fields have some properties that do not have electric currents. Magnetic fields travel through membranes without distortion because the respective permeability is essentially the same as free space [93], whereas electric fields strongly depend on the dielectric properties of the tissue. Therefore, biomagnetic fields produced by neurons and astrocytes are only attenuated by the distance between each other (Figure 3).

**Figure 4:**
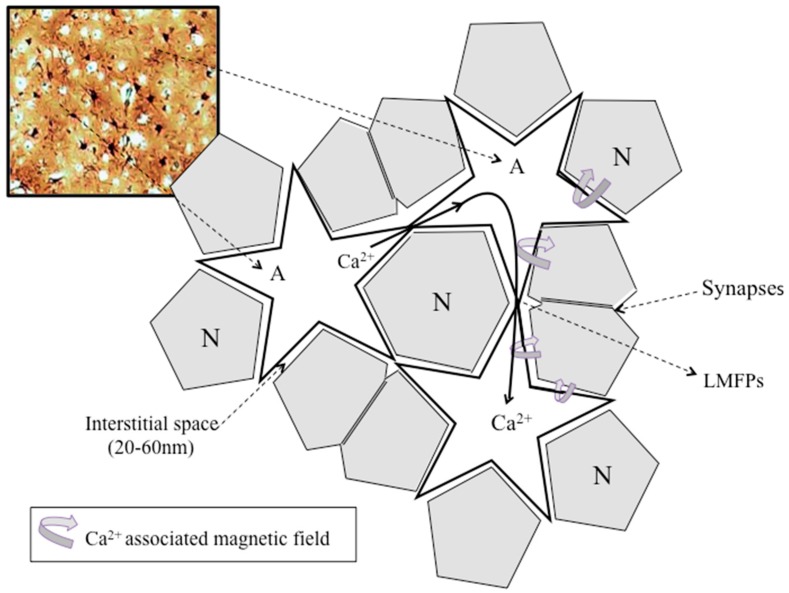
Schematic representation of neurons (N) and astrocytes (A) in layer 2–3 of the human neocortex. Astrocytes are stellated white cells connected by gap junctions. The astroglial matrix surrounds neuronal axons and dendrites. The interstitial space is near virtual (20 to 62 nm width) in physiological conditions. The addition of synaptic currents, action potential currents, and astroglial ionic currents generate local field potential (LFP) in the intercellular space with their corresponding local magnetic field potential (LMFP). Ca^2+^ waves generate current gradients into the astroglial network with their inherent bioelectric and biomagnetic fields that modulate adjacent neuronal behavior by influencing the LFPs and LMFPs.

Since ionic flows and the corresponding biomagnetic fields are likely most significant inside neurons, these magnetic fields pass through neuronal membranes without attenuation, contributing to the interstitial LMFP at a specific point. Bioelectric currents are scalar fields, whereas local biomagnetic fields are vectorial, supplying information on the amplitude and direction of the current sources. Thereby, the measurement of LMFP allows the precise localization of the source of cellular activity at a given time in the 3D volume of the cerebral neocortex (Figure 3). Using new developed “magnetrodes,” it has been possible to measure biomagnetic fields in the neuropil of live cats generated in response to visual stimulation. These magnetic fields were on the order of few nanoteslas [93], which is in agreement with previously calculated magnetic fields having taken into account the nanostructure of the neocortex [24]. Georgiev [94] calculated a magnetic field strength of around 2 × 10^−7^ T in human myelinated axons of 25 × 10^−6^ m having a current intensity [I] of 1 × 10^−6^ A [95], an effective magnetic permeability μ_eff_ = 10, and a magnetic permeability of the vacuum [μ_0_] of 4π × 10^−7^ H/m. However, nonmyelinated axons in the neocortex range in diameter from 0.3 to 1.2 μm, which give a theoretical axonal magnetic field strength adjacent to the neuronal membranes of around 10^−7^ T when a biologically realistic current [I] of 1 μA is used for neocortical neurons. Thus, there is high concordance between the amplitude of the magnetic fields of neurons obtained by experimental [84,93] and theoretical [94] approaches, supporting the idea that biomagnetic fields generated into the neocortex have enough amplitude to be biologically active, having local effects through the modulation of LMFPs [23,24,96]. In addition, the biomagnetic fields generated by Ca^2+^ ionic currents in the astroglial syncytium may likely modulate feedback on the activity of neighboring neurons through the modulation of extracellular LMFPs [23,46] (Figure 4).

### 4.1. LFP, LMFP, and Ca^2+^-Associated Astroglial Biomagnetic Field

Cognitive functions are associated with complex neocortical activity resulting from different patterns of synchronization of thousands of neurons that work in a sophisticated and organized manner using oscillations with specific frequency bands [56]. These cortical oscillations are the result of convergent molecular and electrophysiological mechanisms that critically merge from neuron–astroglial interplay [56]. All these mechanisms can be finally dependent on the highly excitable property of neurons and astrocytes into the neocortex that continuously receives and emits information coherently.

Increasing evidence supports the view that ephaptic interactions play a central role in cognitive faculties [7,65,97,98], mediated by the modulation of adjacent cell activities [99,100,101,102], and LFP interactions across extracellular spaces facilitating neuron–glia communication [49,51,66,98,103,104]. The role of ionic currents on bioelectromagnetic fields was studied in the giant squid axon [105] using complex mathematical algorithms [106,107]. However, the inclusion of Ca^2+^ currents through voltage-gated channels and gap junction among astrocytes increases the complexity of the theoretical approaches [108,109]. Since spontaneous neocortical activity in adult rats propagates horizontally, radially, and isotropically across the cortical layers with a median speed of 35 mm/ms and a peak frequency of around 2.5 Hz [110], we propose that this “spontaneous” neocortical activity, which is characteristic of slow-wave sleep in the mammalian neocortex, is induced back by the intracortical magnetic interaction belonging from neighboring astroglia [10,23,24] (Figure 3 and Figure 4). Thus, the term “spontaneous” might not be adequate, since calcium waves into the astroglia may likely induce ephaptic interactions that modulate random neuronal activity in some layers of the cortex, as has been demonstrated recently [110]. Indeed, based on real EEG recording, a top-down model describes the effects of shallow magnetic fields on the momentum of Ca^2+^ ions at a neocortical level, including the synchronization effects on neurons. These effects of columnar magnetic vector potential on Ca^2+^ ions support the idea that large-scale, top-down processes may modulate classic molecular bottom-up mechanisms implicated in neuronal firing, oscillations, and synchrony [108,109]. As described by the statistical mechanics of neocortical interactions (SMNI) approach, information processing belonging from columnar firings can result in a vector potential (A) that influences the molecular Ca^2+^ momentum (p). The vector potential A is in the same direction as the current flow of Ca^2+^ waves closely aligned to the direction perpendicular to neocortical layers, especially during strong collective EEG activities like specific attention tasks supporting short-term memory. Therefore, larger-scale top-down neocortical information processing may influence molecular-scale bottom-up changes (Ca^2+^ transients) by neuronal synchronization [108,109]. Although the integration of biophysical, biochemical, anatomical, and bioelectromagnetic mechanisms into a model of memory remains to be solved, our hypothesis suggests that information is first encoded in the bioelectromagnetic interplay between neurons and neighboring astrocytes. In this way, changes in the bioelectromagnetic patterns in neocortical layers and columns will result in short-term storage of information. The subsequent molecular (neurotransmitter) and anatomical (synaptic plasticity) mechanisms would eventually produce stable modifications in neuronal circuitry and their related astroglia matrix, supporting long-term memory. Therefore, information must be stored in a distributed form throughout the neocortex, depending on the regional attribution of the predominant sensorial inputs of data and their relationship with other anatomical cerebral areas.

Since astroglial syncytial isopotentiality most likely occurs uniformly across the brain, including spinal cord [111], I conjecture that astrocytes may communicate throughout the CNS, allowing a permanent bioelectric transmission of information by ionic transients.

Physiological LFPs in the neocortex, which depend on its nanostructure, enhance spiking synchrony of pyramidal neurons for slow extracellular oscillations, mainly fewer than 8 Hz (delta and theta bands) [66]. It was suggested that for slow frequencies, neuronal membranes behave following a simple linear amplification model with a constant firing threshold [112]; however, we think the ultrastructural organization and the high inhomogeneity of the interstitial space in the cerebral cortex support that LMFPs (interstitial biomagnetic field gradients) likely contribute to the ephaptic coupling of neurons at very low oscillations (Figure 4). Accordingly, a very sensitive and high spatial and temporal resolution widefield imaging method [84] showed that electric fields around pyramidal neurons are about 2 mV/mm at 10^−6^ m, which corresponds to 5nT magnetic field strength, in agreement to those recently measured [93], suggesting that LMFPs as low as a few nT may contribute to the ephaptic coupling of cortical neurons.

Like in other cell types, intracellular Ca^2+^ oscillation can be induced by Ca^2+^ influx through channels/ionotropic receptors or released from intracellular organelles, playing a myriad of cellular functions, which have been recently reviewed [113,114]. Different pathways of intracellular Ca^2+^ oscillations can be elicited in astrocytes [114]. In general, intracellular astrocyte Ca^2+^ signals are localized in microdomains in large branches 1) as spontaneous Ca^2+^ transients (without any external stimulation); 2) as neuronally mediated Ca^2+^ transients in the hippocampus; or 3) as local spontaneous Ca^2+^ waves encompassing major branches and occasionally astrocyte soma. Another type of Ca^2+^ transient is a global wave encompassing the entire astrocytes, including their soma, triggered by intense bursts of action potentials in the CA3 area. Besides, Ca^2+^ waves occur in the end-feet around the microvasculature. The sixth type is a widespread Ca^2+^ wave mediated by the in vivo release of neuromodulators. The development regulates the seventh Ca^2+^ transients between different astrocyte compartments. The eighth type of Ca^2+^ signal is mediated by the opening of the permeability transition pore in mitochondria. Finally, a long-lasting (~70 s) but highly localized Ca^2+^ signal called twinkles has been recorded in cortical astrocyte branchlets in vivo (reviewed in [114]). Despite the complexity of Ca^2+^ observed in different in vitro, in vivo, and computational models [113], glutamate neurotransmission seems to increase the astrocytic Ca^2+^ levels yielding Ca^2+^ wave propagation between astrocytes that may allow Ca^2+^ increase in neighboring neurons (reviewed in [113]). Therefore, astroglial Ca^2+^ transients are used by neurons to sense ongoing neural activities, including distant network coupling.

We have proposed that the astroglial network has a critical role in the ephaptic coupling of neurons through Ca^2+^-associated biomagnetic fields [24]. Ca^2+^ dynamics into astrocytes increases both spontaneously and in response to neuronal activity [115,116,117,118]. Though controversial results about the role of astroglial Ca^2+^ dynamics on neuronal excitability exist [119,120,121], some studies have demonstrated large-scale Ca^2+^ waves propagating at 61 μm/s among astrocytes, inducing a reduction in the LFPs and neuronal activities in the hippocampus [122]. These large-scale Ca^2+^ transients were associated with the most substantial reduction in the neuronal activity at infralow frequencies (<0.5 Hz). Besides, other types of astrocytic Ca^2+^ waves exist, which propagate around 10–20 μm/s in brain slices and cultures [123], and at 30–40 μm/s in spreading depression [124]. The diversity of Ca^2+^ waves through the astroglial network suggests complex effects on the LMFPs and likely physiological functions on the ephaptic coupling of neurons (Figure 3 and Figure 4).

Using 3D in vitro constructs, Saito et al. showed that the astroglial network modulates spontaneous neuronal electrical activities such as periodic synchronized bursting patterns, showing that the induction of intracellular Ca^2+^ transients in astrocytes correlated with the synchronized bursting activities in the neuronal layers [125]. Although the authors suggested the possibility of gliotransmitter release from the astroglial syncytium in explaining these effects [26,27,126,127], we suggest that LMFPs associated with Ca^2+^ waves into the astroglial syncytium can modulate neural dynamics, contributing to neural coupling via field effects [9,10,23,24,96,109,128].

In order for the Ca^2+^-associated biomagnetic fields generated in the astroglial matrix to participate in the ephaptic coupling of neighboring neuronal populations, any Ca^2+^ waves must travel through the astroglial matrix with little interference from other ions, which is facilitated by the isopotentiality of the astroglial matrix. The timescale of astrocyte Ca^2+^ signaling varies between hundreds of milliseconds (ms) to several seconds (s), which is orders of magnitude larger than any neuronal event such as action potentials (few ms) or postsynaptic potentials (tens of ms). However, the average human reaction times to different stimulus varies between 150 to 250 ms [74] and, for example, a conscious intention to move takes around 1.5 s [74], which are more in agreement to the timescale of astroglial Ca^2+^ signaling. Accordingly, a single astrocytic domain can envelope hundred to thousands of synapses and, therefore, Ca^2+^-associated transients in astroglial networks may potentially integrate the millions of neuronal synapses represented in astroglial biomagnetic fields that self-organize into dynamic physical structures [92] with the ability to modulate, in return, neuronal firing activities. Indeed, the spatiotemporal properties of Ca^2+^ waves in the astroglial network have been considered a guiding template for the coupling of neuronal activities [74]. The astroglial matrix may then function as a multichannel recording of the biomagnetic signals generated by neuronal circuits sharing the same astroglial scaffold that allows the computation of the functional connectivity of thousands of neurons, contributing back to the ephaptic coupling of neurons [23,24] (Figure 3).

### 4.2. Transcranial Electromagnetic Stimulation: Role of Astroglial Isopotentiality

It was thought that tiny magnetic and electric fields were not capable of affecting neuronal activity because they were not able to trigger or suppress neuronal action potentials. However, in vitro and in vivo studies have demonstrated that small electric [65,112,128,129,130] and magnetic [131] fields can modify diverse brain functions. Transcranial electric and magnetic stimulation with fields similar to those that occur endogenously show broad therapeutic and cognitive effects in experimental and clinical studies, including effects on memory encoding, consolidation, and perception [14,65,132,133,134,135]. Indeed, the application of slow AC fields (<1 Hz) during non-REM sleep in humans showed increased cortical slow oscillations and retention in a declarative memory task [133].

Transcranial electric and magnetic field stimulation has effects on the neuronal membrane polarization and ionic currents [136], but other biological effects include changes in blood–brain barrier permeability, vasodilation, effects on neurotransmitter activity, neuronal metabolism, and protein signaling [14]. Transcranial magnetic stimulation generates a magnetic field that induces an electric field and their corresponding current density fields in the brain [14], but the specific mechanisms that mediate their clinical and behavioral effects are incompletely understood [137].

Direct or alternating transcranial current stimulation induces weak extracellular bioelectric fields inside the brain that are similar to endogenous generated fields (around 1–2 mV/mm) [66,138]. These extracellular fields induce currents through neuronal membranes that depend on cell morphology and spatial orientation [138]. It seems that at the soma of pyramidal neurons, the polarization is stronger for fields oriented parallel to the somatodendritic axis, decreasing with the field frequency [138]. However, recent computational models indicate that there is a close frequency resonance around 10–20 Hz in the field sensitivity of apical dendrites that is absent in the soma and basal dendrites [138]. This resonance sensitivity of pyramidal neurons could be due to hyperpolarization-activated cation currents [138]. The polarization of apical dendrites due to alternating current stimulation may modulate Ca^2+^ spikes preferentially generated in this apical arbor [138]. These findings suggest that direct current and very slow frequency fields may mainly polarize the soma and basal dendrites of pyramidal neurons while alternating current stimulation in the range of 10–20 Hz may polarize principally apical dendrites [138]. In agreement, experimental results show that the ephaptic effect is determined by the gradient of the Ve rather than by the magnitude of the Ve itself, suggesting that is the magnetic field gradient rather than its intensity the factor that mainly affects the electrophysiological modulation of neuronal activities [139,140]. These results are consistent with the finding that extracranial variable magnetic fields as low as 10^−6^ T can influence semantic memory in healthy subjects [132].

Astrocytes may participate in the cellular effects of transcranial magnetic field stimulation [141] since they have a critical role in extracellular K^+^ homeostasis and high sensitivity to slight extracellular K^+^ changes [35,51], showing broad isopotentiality among distant regions in the neocortex [77,78]. Moreover, the injection of current into individual astrocytes may induce significant increases in extracellular K^+^ levels [142], supporting the concept that LFPs can be modulated as the result of transcranial field stimulation [143]. Interestingly, the application of 1 Hz transcranial electric fields showed the synchronization of neurons in rat neocortex and the coupling of spiking neurons from distant regions of the cortex in vivo from an unknown origin [144]. Can these effects be related with the astroglial transients of ions? Since Ca^2+^ ionic currents through gap junctions allow widespread distribution of Ca^2+^ signals through the whole astroglial network [122,145] and astrocytes mediate the modulation of diverse neuronal activities [146,147], we propose that magnetic fields associated with Ca^2+^ transients may contribute to neuronal synchronization in response to transcranial field stimulation [24]. In agreement, physiological changes in astrocytic membrane potential in response to neuronal activity [148] are in the range of those estimated for transcranial direct current stimulation (tDCS) [149]. Furthermore, tDCS generates large-amplitude and synchronized Ca^2+^ transients in astrocytes, suggesting an essential role of astrocytic Ca^2+^ waves in the tDCS-induced enhancement of the visual evoked potential in the primary visual cortex of mice [150].

Finally, low transcranial static magnetic field stimulation (tSMS) can decrease cortical excitability in the human motor cortex [151], suggesting that changes in neuronal excitability induced by tSMS would, at least partially, depend on direct magnetic field effects. A possible contributing factor is that tSMS modifies the vector potential of Ca^2+^ ions throughout the astroglial matrix [109], resulting in a change in the magnetic field gradient, driving the flow of Ca^2+^ ions far away from the site of tSMS application. Indeed, astroglial Ca^2+^ waves may be a source as well as a mediator of the bioelectric and biomagnetic field in the neocortex, contributing to its physiological behavior [96]. Therefore, the spontaneous neuronal activity in the cerebral cortex that physiologically displays a large variety of dynamics associated with different cognitive faculties, fluctuating between synchronized activities associated with criticality and desynchronized states associated with subcriticality [79,152,153], may be explained by the proposed neuron–astroglial biomagnetic dialogue [23,24]. A recent “reverberating” model of cortical spike propagation better predicts the behavior of collective neuronal dynamic than previous models [154], suggesting that “criticality” and “asynchronous–irregular” regimes over- and underestimate the real network responses in the neocortex. A significant consequence of the reverberating model is that about 98% of the neuronal network activity is generated by recurrent excitation, and only the rest 2% originates from inputs or spontaneous activities depending on the cortical brain area, cortical layer, and cognitive state [154], which would fit better with our hypothesis.

## 5. Ongoing Challenges

The electromagnetic activity of the brain can be recorded at different levels of organization from the microscale (single ion channel and intracellular recordings) to the macroscale (EEG and MEG), and also at mesoscale level (LFP and LMFP). According to the theory of electromagnetism, any electrical current generates a magnetic field, but only at the macroscale level, and we can use the superconducting quantum interference device (SQUID) magnetometers to detect the macroscale MEG measurement. Therefore, MEG cannot distinguish LMFPs from active neuronal populations [93].

Recent experimental approaches have shown that brainwave magnetic resonance (BMR) allows the visualization of coherent sources of collective spiking neurons using direct SQUID-based micro-Tesla NMR [155] to resonate proton spins around the coherent brain wave excitation of a specific frequency band. The cyclic excitation of synchronic spiking neurons generates electric currents with their associated alternating magnetic fields, whose frequency corresponds to a specific frequency in the theta (4–7 Hz), alpha (8–12 Hz), beta (13–30 Hz), or gamma (30–200 Hz) band. These oscillations contain and transport information of corticocortical as well as corticothalamic functional networks that participate in perception, attention, and memory, and they were strong enough to be measured by EEG or MEG. Interestingly, BMR measurements can record both the tangential and radial components of the neuronal current, while the MEG is blind to the radial component and the EEG is blind to the tangential one [155]. Indeed, the strengths of the magnetic fields corresponding to those frequencies were around 0.1–5 × 10^−6^ Tesla [155]. This technical advancement can discriminate between the NMR signals from the direct biomagnetic signal associated with neuronal coupling, and it has the potential to resolve some central questions in cognitive neuroscience.

Other recent approaches can detect biological magnetic fields at the mesoscale level, using the nitrogen-vacancy (NV) quantum defects in diamond to measure the magnetic field produced by action potentials in the squid and worm giant axons [156]. Besides, this technique can measure local magnetic fields at room temperature [156]. The authors used the skeletal soleus muscle to measure the synchronicity of the electrical activity among the muscle fibers. Skeletal muscles have a fascinating structure composed by groups of parallel excitable muscular cells, innervated at their center by a single excitatory cholinergic synapse. The synaptic transmission is optimal since each single nerve induces postsynaptic action potential through synapses that are well aligned at the center of the muscle, resulting in the coupling among all the muscular fibers working as a syncytial and parallel 3D structure that maximizes the summation of synchronized magnetic fields associated with action potentials across fibers [156]. These elegant experiments can be an inspiration to investigate the role that the astroglial matrix plays in the behavior of the cerebral cortex as well as its involvement in cognitive faculties.

## 6. Conclusions

In recent years, the important role of astrocytes in the mammalian neocortical structure and function has been increasingly demonstrated. In addition to providing structural, metabolic, and ionic homeostatic support to neurons, astroglia play a central role in neuromodulation, synaptic plasticity, and computation that contribute to cognitive faculties. The role of astrocytes in synaptic communication and remodeling is now well recognized, but the widespread role of astroglia in ephaptic neuronal coupling and information processing has been scarcely investigated nor readily modeled.

This paper reviews the scientific data supporting the probable contribution of astrocytes on neuronal modulation by ephaptic interaction, taking into account the structural organization of the neocortex. The isopotentiality of the astroglial matrix allows for continuous equalization of the neocortical oscillations and supports the notion that Ca^2+^-associated biomagnetic fields in astrocytes likely contribute to the coupling of cortical neurons by direct magnetic induction of extracellular LMFPs. Even more important is the possibility that bidirectional biomagnetic dialogue may continuously support the codification of information that would better explain the cognitive functions of the CNS. Further studies using some of the experimental approaches reviewed in this paper will confirm or refute the present hypothesis, including the refinement of transcranial electric and magnetic field stimulating methods, which are revolutionizing the therapy of many neurological and psychological disorders, as well as our understanding of how the brain process and integrates information. We propose that neuronal and astroglial biomagnetic fields generated inside the neocortex are not epiphenomena since they are intrinsically connected to current densities, LFPs, and LMFPs, as has been demonstrated with new technological approaches supporting bidirectional, bioelectromagnetic neuron–astroglial communication in the neocortex [23,24]. Indeed, the bioelectric and biomagnetic fields may causally precede any current and potential secondarily generated in tissues [96].
